# A Clinical Risk Prediction Tool for Peritonitis-Associated Treatment Failure in Peritoneal Dialysis Patients

**DOI:** 10.1038/s41598-018-33196-2

**Published:** 2018-10-04

**Authors:** Surapon Nochaiwong, Chidchanok Ruengorn, Kiatkriangkrai Koyratkoson, Kednapa Thavorn, Ratanaporn Awiphan, Chayutthaphong Chaisai, Sirayut Phatthanasobhon, Kajohnsak Noppakun, Yuttitham Suteeka, Setthapon Panyathong, Phongsak Dandecha, Wilaiwan Chongruksut, Sirisak Nanta, For the Thai Renal Outcomes Research (THOR) Investigators, Yongyuth Ruanta, Apichart Tantraworasin, Uraiwan Wongsawat, Boontita Praseartkul, Kittiya Sattaya, Suporn Busapavanich

**Affiliations:** 1 Department of Pharmaceutical Care, Faculty of Pharmacy, Chiang Mai University, Chiang Mai, 50200 Thailand; 2 Pharmacoepidemiology and Statistics Research Center (PESRC), Faculty of Pharmacy, Chiang Mai University, Chiang Mai, 50200 Thailand; 3 Ottawa Hospital Research Institute, Ottawa Hospital, Ottawa, Ontario, K1H 8L6 Canada; 4 School of Epidemiology and Public Health, Faculty of Medicine, University of Ottawa, Ottawa, Ontario, K1G 5Z3 Canada; 5 Institute of Clinical and Evaluative Sciences, ICES@UOttawa, Ottawa, Ontario, K1Y 4E9 Canada; 6 School of Pharmaceutical Sciences, University of Phayao, Phayao, 56000 Thailand; 7 Division of Nephrology, Department of Internal Medicine, Faculty of Medicine, Chiang Mai University, Chiang Mai, 50200 Thailand; 8 Kidney Center, Nakornping Hospital, Chiang Mai, 50180 Thailand; 9 Division of Nephrology, Department of Internal Medicine, Prince of Songkla University, Hat Yai, Songkhla, 90110 Thailand; 10 Department of Surgery, Faculty of Medicine, Chiang Mai University, Chiang Mai, 50200 Thailand; 11 Mae Sai District Hospital, Mae Sai, Chiang Rai, 57130 Thailand

## Abstract

A tool to predict peritonitis-associated treatment failure among peritoneal dialysis (PD) patients has not yet been established. We conducted a multicentre, retrospective cohort study among 1,025 PD patients between 2006 and 2016 in Thailand to develop and internally validate such a tool. Treatment failure was defined as either a requirement for catheter removal, a switch to haemodialysis, or peritonitis-associated mortality. Prediction model performances were analysed using discrimination (C-statistics) and calibration (Hosmer-Lemeshow test) tests. Predictors were weighted to calculate a risk score. In total, 435 patients with 855 episodes of peritonitis were identified; 215 (25.2%) episodes resulted in treatment failure. A total risk score of 11.5 was developed including, diabetes, systolic blood pressure <90 mmHg, and dialysate leukocyte count >1,000/mm^3^ and >100/mm^3^ on days 3–4 and day 5, respectively. The discrimination (C-statistic = 0.92; 95%CI, 0.89–0.94) and calibration (*P* > 0.05) indicated an excellent performance. No significant difference was observed in the internal validation cohort. The rate of treatment failure in the different groups was 3.0% (low-risk, <1.5 points), 54.4% (moderate-risk, 1.5–9 points), and 89.5% (high-risk, >9 points). A simplified risk-scoring scheme to predict treatment failure may be useful for clinical decision making regarding PD patients with peritonitis. External validation studies are needed.

## Introduction

Over the past decade, treatment practice and technical improvements in peritoneal dialysis (PD) have substantially decreased PD-associated adverse outcomes, allowing end-stage kidney disease (ESKD) patients to be sustained on PD programs for longer periods^1,2^. Unfortunately, PD-related peritonitis still remains one of the most serious complications in PD patients worldwide, which results in significant morbidity, mortality, and health care expenditure^1,3–5^. It also considerably limits its choice as a renal replacement therapy modality among ESKD patients^6^. Ongoing PD-related peritonitis and a failure to respond to treatment can lead to structural and functional alterations of the peritoneal membrane, leading to membrane malfunction possibly as a consequence of encapsulating peritoneal sclerosis^7,8^. Importantly, PD-related peritonitis has been well known to contribute up to 18–20% of the technical failure of PD necessitating a subsequent switch to long-term haemodialysis^1,4,9^.

Numerous studies have attempted to describe the risk of peritonitis-related treatment failure. However, these studies were generally designed to identify risk factors that affect treatment outcomes^10–15^. Current risk evaluation using the patient’s clinical status, namely the disease severity score, has been developed to assess the severity symptoms at the onset of peritonitis and is widely used in the paediatric PD population^16–19^. Yet, this approach has been limited by poor reporting standards in terms of the predictive performance and the failure to validate its utilisation in the adult PD population^20^.

Although predictors of treatment failure in patients with PD-related peritonitis are well recognised, surprisingly no risk prediction tool has been established. Critically, clinicians must make ad-hoc decisions based on clinical experience, which may be insufficient to classify patients with regard to PD technical failure and mortality risk. Therefore, the use of acknowledged risk factors to predict the probability peritonitis-related treatment failure is helpful for both clinicians and patients, as the prediction would minimise subsequent complications as well as the costs of health care services. Given the poor outcome of PD-related peritonitis, the early detection of information relevant to the treatment failure may be confer an advantage in the planning treatment intervention; such as early catheter removal and device insertion for temporary haemodialysis.

To guide decision making in the clinical setting and improve the quality of PD programs, we sought to develop and internally validate a simplified risk prediction tool based on readily available and routinely obtained predictors that could be used to predict treatment failure during an episode of PD-related peritonitis.

## Results

### Cohort description

From 1 January 2006 to 31 December 2016, 1,025 PD patients were screened. Of these, a total of 1,000 episodes (0.39 episodes per patient-year) met the criteria for PD-related peritonitis, and 145 episodes were excluded (Supplementary Figure S1). Therefore, 855 episodes of peritonitis in 435 PD patients were included in the analysis: 640 (74.8%) in whom treatment was successful and 215 (25.2%) classified as treatment failure. Forty-seven (5.5%) patients died due to PD-related peritonitis. Peritonitis characteristics were categorised according to the outcomes of treatment (Table 1). The patients had a mean age at the peritonitis presentation of 59.6 ± 13.0 years. The majority were male (52.0%) and undergoing continuous ambulatory peritoneal dialysis (95.9%). Of the 855 episodes, the majority of causative organisms were gram-positive bacteria (38.9%), and 36.3% of the cohort had negative culture results. Missing values for each variable ranged from 0.0 to 6.9%; no variable had more than 20% of data missing.

**Table 1:** Peritonitis Characteristics According to the Outcomes of Treatment.

Characteristics	Overall (n = 855 Episodes)	Missing data	Outcomes of Treatment		*P* Value
			Success (n = 640 Episodes)	Failure (n = 215 Episodes)	
**Socio-demographic Factors**					
Gender, male	445 (52.0)	0 (0.0)	337 (52.7)	108 (50.2)	0.581
Age (years), mean ± SD; range	59.6 ± 13.0	0 (0.0)	59.3 ± 12.1	59.6 ± 13.0	0.773
Primary cause of ESKD		0 (0.0)			
Hypertensive nephrosclerosis	273 (31.9)		198 (30.9)	75 (34.9)	0.609
Diabetic nephropathy	332 (38.8)		250 (39.1)	82 (38.1)	
Glomerulonephritis	44 (5.2)		32 (5.0)	12 (5.6)	
Others/unknown	206 (24.1)		160 (25.0)	46 (21.4)	
Comorbidity					
Hypertension	740 (86.6)	0 (0.0)	546 (85.3)	194 (90.2)	0.083
Diabetes mellitus	298 (34.8)	0 (0.0)	211 (33.0)	87 (40.5)	0.048
CAD	61 (7.1)	0 (0.0)	48 (7.5)	13 (6.0)	0.542
CHF	61 (7.1)	0 (0.0)	47 (7.3)	14 (6.5)	0.761
CVD	33 (3.9)	0 (0.0)	21 (3.3)	12 (5.6)	0.151
Malignancy	29 (3.4)	0 (0.0)	24 (3.8)	5 (2.3)	0.389
Chronic hepatitis B	7 (0.8)	0 (0.0)	5 (0.8)	2 (0.9)	1.000
Chronic hepatitis C	16 (1.9)	0 (0.0)	15 (2.3)	1 (0.5)	0.087
Mobility		11 (1.3)			
Independent walker	694 (82.2)		532 (83.8)	162 (77.5)	0.077
Assisted walker	38 (4.5)		24 (3.8)	14 (6.7)	
Chair-bound/bedridden	112 (13.3)		79 (12.4)	33 (15.8)	
Reimbursement scheme		11 (1.3)			
UCS by NHSO	689 (81.6)		510 (80.8)	179 (84.0)	0.543
CSMBS	131 (15.5)		103 (16.3)	28 (13.2)	
SSS/others	24 (2.8)		18 (2.9)	6 (2.8)	
PD modality		0 (0.0)			
CAPD	820 (95.9)		610 (95.3)	210 (97.7)	0.164
APD	35 (4.1)		30 (4.7)	5 (2.3)	
PD with assistance		44 (5.1)			
Alone	428 (52.8)		322 (53.2)	106 (51.7)	0.201
With family	378 (46.6)		282 (46.5)	96 (46.8)	
With others	5 (0.6)		2 (0.3)	3 (1.5)	
Dialysis duration (months), median (min–max)	17.7 (0.0–106.1)	1 (0.1)	17.9 (0.0–106.1)	16.3 (0.2–95.4)	0.968
**Clinical Presentation and Treatment**					
Blood pressure (mmHg), mean ± SD					
Systolic	133.9 ± 24.6	0 (0.0)	135.5 ± 24.2	129.3 ± 25.1	0.001
Diastolic	75.6 ± 15.2	0 (0.0)	76.2 ± 15.0	74.9 ± 16.0	0.058
Fever (°C)		0 (0.0)			
≤38.9	814 (95.2)		615 (96.1)	199 (92.6)	0.043
>38.9	41 (4.8)		25 (3.9)	16 (7.4)	
Abdominal pain	596 (74.9)	59 (6.9)	453 (75.8)	143 (72.2)	0.345
Diarrhoea	229 (27.4)	19 (2.2)	169 (27.0)	60 (28.4)	0.721
Constipation	55 (6.6)	21 (2.4)	43 (6.9)	12 (5.7)	0.631
Catheter leak	22 (2.7)	46 (5.4)	13 (2.2)	9 (4.4)	0.132
Cloudy dialysate fluid	801 (93.7)	0 (0.0)	605 (94.5)	196 (91.2)	0.104
Disease severity score, median (min–max)	1 (0–5)	56 (6.5)	1 (0–5)	1 (0–5)	0.555
Serum albumin, g/dL	3.0 ± 0.4	0 (0.0)	3.0 ± 0.3	2.9 ± 0.5	0.725
Dialysate leucocyte count (/mm^3^), median (min – max)					
On day 1	1,350 (100–81,000)	0 (0.0)	1,150 (100–81,000)	2,070 (100–55,000)	<0.001
On day 3–4	124 (0–27,673)	47 (5.5)	80 (0–8,530)	1,180 (0–27,673)	<0.001
Causative organism					
Gram-positive only^a^	322 (37.7)	0 (0.0)	287 (44.8)	35 (16.3)	<0.001
MRSA	10 (1.2)		3 (0.5)	7 (3.3)	
Gram-negative only^b^	70 (8.2)		51 (8.0)	19 (8.8)	
*Acinetobacter spp*.	33 (3.9)		18 (2.8)	15 (7.0)	
*Pseudomonas spp*.	20 (2.3)		9 (1.4)	11 (5.1)	
Fungi	35 (4.0)		3 (0.5)	32 (14.9)	
Mycobacterial	8 (0.9)		2 (0.3)	6 (2.8)	
Polymicrobial	47 (5.5)		23 (3.6)	24 (11.1)	
Culture negative	310 (36.3)		244 (38.1)	66 (30.7)	
Antimicrobial for empirical therapy		0 (0.0)			
First generation cephalosporin-based regimen	605 (70.8)		474 (74.1)	131 (61.0)	0.001
Glycopeptide-based regimen	156 (18.2)		108 (16.9)	48 (22.3)	
Others regimen	94 (11.0)		58 (9.0)	36 (16.7)	

Note: Data are expressed as frequency (percentage) of patients, unless otherwise indicated.

Abbreviations: APD, automated peritoneal dialysis; CAD, coronary artery disease; CAPD, continuous ambulatory peritoneal dialysis; CHF, chronic heart failure, CSMBS, the Civil Servant Medical Benefit Scheme; CVD, cerebrovascular disease; ESKD, end-stage kidney disease; MRSA, Methicillin-resistant *Staphylococcus aureus*; NHSO, the National Health Security Office; PD, peritoneal dialysis; SSS, the Social Security Scheme; UCS, the Universal Coverage Scheme.

^a^Excluding MRSA.

^b^Excluding *Acinetobacter spp*. and *Pseudomonas spp*.

### Prediction model performance in the derivative phase

The crude odds ratios (ORs) identified 13 candidate predictors with *P* < 0.100 (Supplementary Table S1). The multivariable logistic regression for the predictors and statistics for discrimination and goodness-of-fit for successive models in the derivative phase are illustrated in Supplementary Table S2. Compared to model 3, the concordance statistics (C-statistics) did not improve with the addition of dialysate leukocyte count on day 1 in model 2 (0.94; 95% CI, 0.92–0.96; *P* = 0.160) and did not improve with the inclusion of age and sex in model 1 (0.94; 95% CI, 0.92–0.96; *P* = 0.512). Meanwhile, model 5, which included only 3 variables, gave an acceptable discriminative power (0.76; 95% CI, 0.72–0.80), but the Akaike Information Criterion (AIC) was higher than that for model 3 (*P* < 0.001). The inclusion of the causative organism in model 3 improved both the C-statistics and the AIC for model selection compared with those for model 4 (0.94 [95% CI, 0.92–0.96] vs. 0.92 [95% CI, 0.89–0.94] and 444.05 vs. 482.03, *P* < 0.001, respectively). However, waiting to establish the causative organism may cause a delay in early clinical decision making, particularly in the case of fungal and mycobacterial cultures. Moreover, episodes of fungal peritonitis have a high treatment failure rate in general, usually considered simultaneous catheter removal. Given these results, model 4 was considered for the development of a simple risk-scoring scheme step.

### Risk score derivation and application

**Figure 1:**
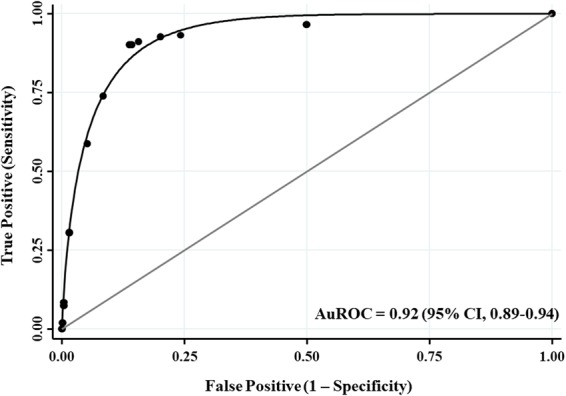
The AuROC Curve and 95% CI of the Prediction Score of Treatment Failure in PD-Related Peritonitis. Abbreviations: AuROC, area under the receiver operating characteristic; CI, confidence interval; PD, peritoneal dialysis.

The numbers of points assigned to each of the four independent risk predictors in accordance with the prediction model 4 are listed in Table 2. Total risk scores ranged from 0 to 11.5 (median 1; ranged [0–11.5]). The C-statistics of the risk score was 0.92 (95% CI, 0.89–0.94; Fig. 1). The *P*-value for the Hosmer-Lemeshow test was 0.930, indicating good agreement between observed outcome and predicted risk score. The risk score performance is shown in Supplementary Table S3. Supplementary Figure S2 shows good concordance between scored predicted and observed risk of treatment failure.

**Table 2:** Prediction Model and Item Scoring Scheme for Treatment Failure in PD-Related Peritonitis.

Predictors	Adjusted OR (95% CI)	*P* Value	β-Coefficients (SE)	Transformed Score	Assigned Score
**Diabetes mellitus**					
No	1.00 (Reference)	—	—	0	0
Yes	1.81 (1.09–3.01)	0.022	0.59 (0.26)	1	1
**Systolic BP** <**90** **mmHg**					
No	1.00 (Reference)	—		0	0
Yes	4.36 (1.72–11.09)	0.002	1.47 (0.48)	2.49	2.5
**Dialysate leucocyte count on day 3–4** >**1,000/mm**^**3**^					
No	1.00 (Reference)	—	—	0	0
Yes	2.52 (1.50–4.23)	<0.001	0.92 (0.26)	1.56	1.5
**Dialysate leucocyte count on day 5** >**100/mm**^**3**^					
No	1.00 (Reference)	—	—	0	0
Yes	43.64 (25.69–74.16)	<0.001	3.78 (0.27)	6.41	6.5

Abbreviations: BP, blood pressure; CI, confidence interval; OR, odds ratio; PD, peritoneal dialysis; SE, standard error.

**Figure 2:**
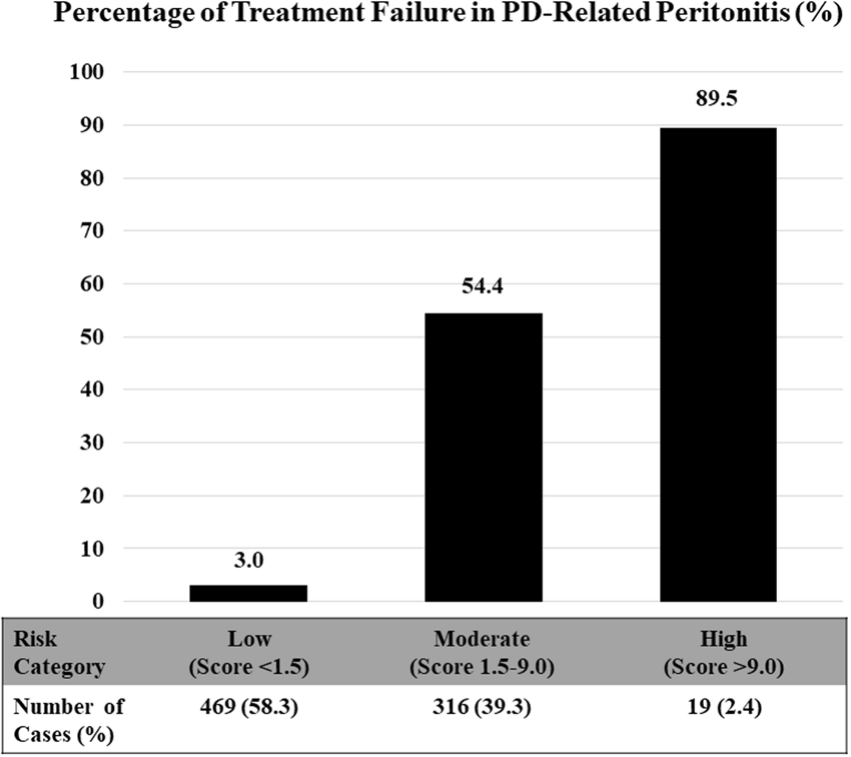
Treatment Failure in PD-Related Peritonitis According to the Simplified Risk Category (804 Episodes). Abbreviations: PD, peritoneal dialysis.

On the basis of clinical practicability and simplicity, the total scores were classified as low (<1.5), moderate (1.5–9), and high risk (>9) for treatment failure with the positive likelihood ratio (LHR+) of 0.09 (95% CI, 0.05–0.15), 3.54 (95% CI, 3.03–4.12), and 25.16 (95% CI, 5.86–107.98), respectively (Table 3). The observed treatment failure rate for the simplified risk strata increased from 3.0% (95% CI, 1.6%-5.0%) in the low-risk group and 54.4% (95% CI, 48.8%–60.0%) in the moderate-risk group to 89.5% (95% CI, 66.9%–98.7%) in the high-risk group (Fig. 2).

**Table 3:** Risk Category among Patients with Treatment Failure in PD-Related Peritonitis (804 Episodes).

Risk Category	Treatment Failure		LHR+ (95% CI)	*P* Value
	No (n = 601 Episodes): n (%)	Yes (n = 203 Episodes): n (%)		
Low (<1.5)	455 (97.0)	14 (3.0)	0.09 (0.05–0.15)	<0.001
Moderate (1.5–9)	144 (45.6)	172 (54.4)	3.54 (3.03–4.12)	<0.001
High (>9)	2 (10.5)	17 (89.5)	25.16 (5.86–107.98)	<0.001

Abbreviations: CI, confidence interval; LHR+, likelihood ratio of positive; PD, peritoneal dialysis.

### Internal validation

With bootstrapping, the C-statistic of the risk score was 0.91 (95% CI, 0.86–0.96; Supplementary Figure S3), which is very similar to the derivation set (the degree of optimism or slope shrinkage was 0.007). For calibration, no statistically significant difference was observed (*P*-value for Hosmer-Lemeshow statistic = 0.889). The estimated Somers’ D correlation coefficients for the derivative model and bootstrap model were 0.832 and 0.822, respectively (difference 0.035; 95% CI, 0.010–0.050). Given these results, our risk score allows us to distinguish fairly accurately between patients who were in the treatment failure group from those who were being treated in the PD population whose characteristics are similar to our cohort.

### Sensitivity analyses

For sensitivity analyses, using the multiple imputation analysis, recalculation of peritonitis-related treatment failure definition and restricting the analysis by subset of causative organism in accordance with the analytical methods, did not alter the prediction model performances. The discrimination (C-statistic) ranged from 0.90 to 0.94, and all *P*-values for Hosmer-Lemeshow statistic were >0.05 (Supplementary Table S4).

## Discussion

We developed and internally validated a risk-scoring scheme for stratifying PD patients as low, moderate, and high risk for treatment failure after an episode of peritonitis. The simplified risk-scoring scheme revealed an excellent performance of the model prediction, in terms of discrimination and calibration.

Several existing predictors of treatment failure in patients with peritonitis were examined (Supplementary Table S1) but were not retained in the final model. In addition, during the model selection process, we excluded the causative organism risk predictor because the time delay imposed by organism identification may postpone timely decision making. Subsequently, four predictors of treatment failure among patients with peritonitis were selected. These included diabetes, systolic blood pressure of <90 mmHg at presentation, and a dialysate leukocyte count of >1,000/mm^3^ and >100 mm3 on days 3–4 and day 5, respectively. These factors have been previously recognised to be predictors of peritonitis-related treatment failure^10,12,13,15,20^. Not surprisingly, dialysate leukocyte count, especially on day 5, is the strongest predictor (responsible for 88% of predictive model), which reflects the response to treatment in PD-related peritonitis^13,21^.

**Figure 3:**
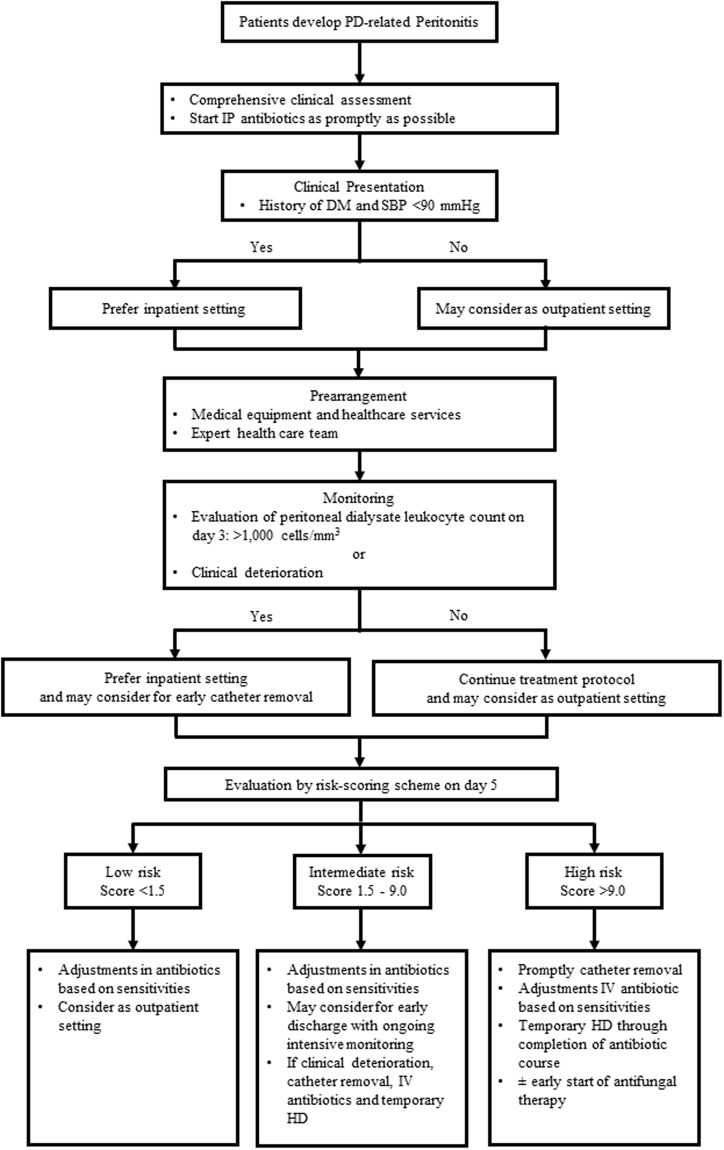
Proposed Risk-Stratification Algorithm for Clinical-Decision Making on the Appropriate Strategy of Care for PD-related Peritonitis, According to Their Level of Risk of Treatment Failure. Abbreviations: DM, diabetes mellitus; HD, haemodialysis; IP, intraperitoneal; IV, intravenous; PD, peritoneal dialysis; SBP, systolic blood pressure.

To improve the clinical utilisation of this risk-scoring scheme, we propose a risk-stratification algorithm for clinical-decision making with respect to the appropriate care strategy for PD patients with peritonitis, based on their risk score for treatment failure (Fig. 3). We advocate the use of our risk-scoring scheme as promptly as possible once patients meet the criteria for PD-related peritonitis. This will allow for the most effective allocation of medical equipment, health care services (PD clinics, dialysis-access-surgery lists), and expert health care teams (nephrologist, access surgical team, medical staff).

In recent years, several studies have shown that postponing catheter removal beyond the first week of a peritonitis episode was associated with substantially higher rates of permanent transfer to haemodialysis therapy, as well as an increased risk of morbidity and mortality^14,22–24^. In accordance with the 2016 International Society for Peritoneal Dialysis (ISPD) Peritonitis Guidelines Recommendations^3^, we therefore advise that all patients in the high-risk group (>9 points on day 5) should undergo timely PD catheter removal, owing to the likelihood of experiencing treatment failure. This will help improve long-term technical survival of PD patients. Moreover, for the high-risk group, re-evaluation and refinement of treatment should be considered, including more comprehensive assessment and testing, appropriate adjustments in antibiotic courses or an early start to antifungal therapy, intensive monitoring, and planned early nephrology care as well as other expert health care services.

For the moderate-risk group, early discharge from the hospital with ongoing care in primary care facilities may be considered. However, a plan, in consultation with health care specialty teams, for intensified monitoring, similar to that recommended for the high-risk group should be put in place to encourage rapid and appropriate decision making. For patients deemed to be at a low risk for treatment failure, an outpatient setting with intraperitoneal (IP) antibiotics may be considered, after taking into account factors such as: clinical presentation (severity of sign and symptoms, haemodynamic status), the type of treatment schedule, the availability of facilities to provide IP antibiotics on an outpatient basis, and the ability of the patient and their caregiver^3^.

Nevertheless, it is important to emphasise that our proposed algorithm is anticipated to aid in bedside risk stratification and to anchor decisions on the managed care pathway. Prognosis-based risk stratification should not be used to delay or withhold treatment options, which need to be accompanied by individualised and circumstantial factors. Indeed, it should instead help prioritise the risk of treatment failure and deliver an appropriate strategy of care for PD patients with peritonitis.

To our knowledge, this is the first risk-scoring scheme that has been developed to predict treatment failure among PD patients with peritonitis. From a clinical perspective, it offers a point-based system of prognosis prediction that can be used to enhance decision making. It can help categorise patients with peritonitis at a high risk of treatment failure, who are best placed to gain from early management strategies such as catheter removal together with intensive therapy and monitoring. Additionally, the classification of patients as low risk may help diminish cost and health care expenditure. Besides clinical utility, our risk-scoring scheme can be used as a clinical research tool to identify patients who could particularly benefit from a given intervention or strategy of care.

Published studies suggest a promising role for novel biomarkers as risk predictors of adverse outcomes in patients with peritonitis^25–27^. Research in this field may yield information that can be incorporated into our predictive model to establish the prognostic value of these markers, primarily at the early presentation stage of PD-related peritonitis.

From a methodological point of view, the risk-scoring scheme was established based on a rigorous statistical approach and revealed excellent predictive performance. For clinical practicality, this risk-scoring scheme incorporates information that is routinely and readily available at presentation. This tool will make it possible for a general practitioner, or even a nephrologist, to recognise the risk strata of patients at the bedside. In addition, we have proposed a risk-stratification algorithm that can be used to verify cost-effectiveness analysis which can be incorporated into routine practice along with pragmatic clinical trials.

Our results should be interpreted with caution. Firstly, although the prediction model gives excellent performance and the sample size was statistically adequate, large prospective external validation studies, in different settings, are needed to establish the transportability and generalisability of the prediction model^28,29^.

Secondly, even though we conducted extensive data quality review to ensure accurate data assessment, potential bias owing to missing data, misclassification or under-ascertaining remain possible. However, we attempted to address this limitation using the multiple imputation analysis and restricting the analysis according to the definition of treatment failure and a subset of causative organisms. Our sensitivity analysis indicated that the prediction model performance was robust across different sensitivity analyses.

Finally, the level and intensity of clinical PD practice and the threshold for catheter removal among the three different PD centres may not be uniform over time, which may have influenced the treatment outcome as well as our predictive model. Additionally, the timings of dialysate leukocyte count measurements were inconsistent across the PD centres; prospective studies with regular scheduled measurements (preferably on day 3 and subsequently on day 5) would improve our ability to suggest the best approach for the managed care pathway.

In conclusion, we have developed a simplified risk-scoring scheme which accurately predicts the risk of treatment failure after an episode of PD-related peritonitis, and will be useful as a tool for risk stratification and in clinical decision making. Prospective external validation, including a wider PD population in pragmatic trials as well as the collation of large population-based databases, is needed to evaluate the clinical utility of incorporating this risk-scoring scheme as part of routine PD care.

## Methods

### Study design and patient population

We conducted a retrospective cohort study using data from three large PD centres in Thailand: (i) the Nakornping Hospital, Chiang Mai, the largest PD provider in the province, representing one of the largest PD programs in Northern Thailand; (ii) the Maharaj Nakorn Chiang Mai Hospital, Chiang Mai University, the tertiary referral and university hospital in the Northern Thailand; and (iii) the Songklanagarind Hospital, Prince of Songkla University, a university hospital that was established as one of the pilot PD centres for the “PD First Policy” in Thailand.

Using the local joint registry to obtain data of newly diagnosed PD treated from 1 January 2006 to 31 December 2016, the Thai Renal Outcomes Research-Peritoneal Dialysis (THOR-PD) database, linked and merged datasets including: (i) electronic health records, claims database which contains outpatient and inpatient data; (ii) the Support System Pharmacy Dispensing extract, which contains patient-level detail on medication use; (iii) the PD Patient Care Database, which captures patient-level detail on socio-demographic, clinical characteristics, and long-term care administrative data; and (iv) the Laboratory Support System extract, which includes claims and laboratory results. To ensure accurate data assessment and to limit the quantity of missing data, an external consensus panel of two health information professionals, well trained in kidney disease management, reviewed, verified, and validated the database.

The study was approved by the institutional review boards of Nakornping Hospital, Faculty of Medicine of Chiang Mai University, and Prince of Songkla University. Owing to the retrospective nature of the study and the fact that patient information was de-identified, the requirement for informed consent was waived in all participating facilities. The study protocol was conducted in accordance with the Declaration of Helsinki and reported in compliance with the Transparent Reporting of a Multivariable Prediction Model for Individual Prognosis or Diagnosis statement^30^.

We identified a cohort of ESKD patients who commenced with first-ever PD in an outpatient primary care nephrology clinic between January 2006 and December 2016. Patients eligible for inclusion in this study were (i) aged 18 years or older and (ii) had at least one episode of PD-related peritonitis as defined by the 2016 ISPD recommendations^3^. Patients were excluded if they had either of the following criteria: (i) incomplete follow-up data or (ii) the dialysate leukocyte count had been recorded less than 2 times in the first 5 days.

### Candidate predictors

Potential predictors of peritonitis-related treatment failure were identified based on a comprehensive literature review, and the list of variables was verified by clinicians. These comprised socio-demographic characteristics, including sex, age, aetiology of ESKD, comorbidity, mobility, access to a reimbursement scheme, PD modality, assisted PD, and dialysis duration; clinical presentation and physical examinations at each episode of peritonitis, including blood pressure, symptoms at presentation, disease severity score; and laboratory tests including, dialysate leukocyte count and causative organism; and antimicrobial for empirical therapy^10–15,20^. All candidate predictors were obtained at each episode of PD-related peritonitis through the THOR-PD database.

### Outcome measures

The outcome of interest was peritonitis-related treatment failure, defined as the need to remove the Tenckhoff catheter, a temporary or permanent switch to haemodialysis, or peritonitis-associated mortality within 30 days of an episode of peritonitis^31^. Outcome was ascertained by manually cross-checking the medical chart along with the THOR-PD database.

### Sample size

Sample size estimation was calculated based on (i) various risk factors from previous studies, including older age^14^, aetiology of ESKD^11^, diabetes^13^, duration of PD^13,15^, serum albumin^12^, presence of symptoms (hypotension, diarrhoea, paralytic ileus)^10^, dialysate leukocyte count^12,13,15,20^, and causative organism;^11–14^ and (ii) the precision of the area under receiver operating characteristic (AuROC) statistic of 0.70 to express an interpretable predicted risk score^32^. Moreover, an additional 20% of cases were added to compensate for any missing data. It was estimated that at least 667 episodes of PD-related peritonitis were needed for the present study to ensure a power of 80% and a 0.05 type I error.

### Statistical analysis

All analyses were performed using Stata software version 14.0 (StataCorp LP). Two-tailed test with *P* < 0.05 was considered statistically significant. Predictors with more than 20% of the values missing were excluded from the primary analysis. To address the missing data, however, all other missing values were imputed with a multiple imputation technique^33,34^. For the predictors with less than 10% of missing values, the complete case was accepted and considered in the primary analysis^35^. Nevertheless, the multiple imputation analysis was examined as a sensitivity analysis.

#### Risk score derivation

A combination of clinical guidance and backward selection was performed to determine the candidate predictors. For univariate analysis, candidate predictors with *P* < 0.100 were considered and subsequently included in the multivariable logistic regression analysis to obtain parsimonious models. The collinearity of the final multivariable model was assessed. To develop a simple and clinically applicable tool, we therefore established a consecutive series of models and compared these with more potential predictors to simpler ones.

The C-statistics known as the AuROC curve were computed as measures of discrimination, which refers to the ability of a model to correctly distinguish between those with or without the outcome (treatment failure vs. no treatment failure)^36^. When the C-statistics is greater than 0.7, the model is indicated to having acceptable discriminative ability^32,36^. To compare the different models on a given outcome, the AIC was used to compare the overall model and was deemed fit for consecutive models. The AIC considered both the statistical goodness-of-fit and model simplicity with parsimony of predictors; lower values indicate at better model^37,38^.

To establish a clinical prediction rule, the coefficients from each predictor of the final model were calculated, converted to item scores by rounding to the nearest half (0.5), and summed up to give a total score. The C-statistics measured the predictive ability of a total score indicating peritonitis-related treatment failure. Model calibration was examined using the Hosmer-Lemeshow goodness-of-fit test to compare the agreement between the predicted risks and observed outcome. The predictive model was considered accurate if the *P*-value for the Hosmer-Lemeshow statistic was more than 0.05^36,39^. Finally, a calibration was visualised by plotting the predicted probabilities against observed treatment failure after an episode of PD-related peritonitis.

For clinical practicability, the total risk score was categorised into low, moderate, and high risk of peritonitis-related treatment failure. The LHR+ was estimated to establish the predictive ability of each risk score category. Theoretically, a LHR+ below 0.1 in the low-risk group and above 10 times in the high-risk group are considered as strong evidence to rule in or rule out probabilities of peritonitis-related treatment failure, respectively^40,41^.

#### Internal validation of the risk score

To quantify the potential for overfitting and optimism in model performance, internal validation was performed using a bootstrapping technique with 200 repetitions^42–44^. Compared with the derivation phase, model performance in the validation phase was evaluated using the C-statistics and Hosmer-Lemeshow statistic. The Somers’ D correlation was further estimated to assess the calibration of the model in terms of the agreement between the observed and predicted risk scores of peritonitis-related treatment failure^45^.

#### Sensitivity analyses

To address the robustness of the risk score and evaluate the effect of different clinical care contexts, additional analyses were performed: (i) using the multiple imputation analysis to evaluate the prediction model performance; (ii) assessing discrimination of the predicted risk score when only catheter removal was used to define treatment failure; (iii) excluding episodes with Methicillin-resistant *Staphylococcus aureus* (MRSA) and *Pseudomonas spp*. to account for the risk that these conditions would be considered refractory to treatment; (iv) excluding episodes that had *Mycobacterium* and fungal peritonitis because they have a high treatment failure rate, such infection is an indication for catheter removal according to the 2016 ISPD guidelines^3^; and (v) restricting the analysis to episodes with positive cultures.

## Electronic supplementary material

Supplementary Information
