# Supplementary material for: A Clinical Risk Prediction Tool for Peritonitis-Associated Treatment Failure in Peritoneal Dialysis Patients
Source: Sci Rep. 2018 Oct 4;8:14797. doi: 10.1038/s41598-018-33196-2 (PMC6172229; doi:10.1038/s41598-018-33196-2)
Supplement: Supplementary file 1 — Supplementary Information [file 41598_2018_33196_MOESM1_ESM.pdf]

Supplementary Information

A clinical Risk Prediction Tool for Peritonitis-Associated Treatment Failure in Peritoneal Dialysis Patients

Surapon Nochaiwong[1,2], Chidchanok Ruengorn[1,2], Kiatkriangkrai Koyratkoson, Kednapa Thavorn[2,3,4,5], Ratanaporn Awiphan[1,2], Chayutthaphong Chaisai[1,2], Sirayut Phatthanasobhon[1,6], Kajohnsak Noppakun[2,7], Yuttitham Suteeka[7], Setthapon Panyathong[2,8], Phongsak Dandecha[9], Wilaiwan Chongruksut[2,10], Sirisak Nanta[2,11]; for the Thai Renal Outcomes Research (THOR) Investigators

[1]Department of Pharmaceutical Care, Faculty of Pharmacy, Chiang Mai University, Chiang Mai 50200, Thailand

[2]Pharmacoepidemiology and Statistics Research Center (PESRC), Faculty of Pharmacy, Chiang Mai University, Chiang Mai 50200, Thailand

[3]Ottawa Hospital Research Institute, Ottawa Hospital, Ottawa, Ontario K1H 8L6, Canada

[4]School of Epidemiology and Public Health, Faculty of Medicine, University of Ottawa, Ottawa, Ontario K1G 5Z3, Canada

[5]Institute of Clinical and Evaluative Sciences, ICES@UOttawa, Ottawa, Ontario K1Y 4E9, Canada

[6]School of Pharmaceutical Sciences, University of Phayao, Phayao, 56000, Thailand

[7]Division of Nephrology, Department of Internal Medicine, Faculty of Medicine, Chiang Mai University, Chiang Mai 50200, Thailand

[8]Kidney Center, Nakornping Hospital, Chiang Mai 50180, Thailand

[9]Division of Nephrology, Department of Internal Medicine, Prince of Songkla University, Hat Yai, Songkhla 90110, Thailand

[10]Department of Surgery, Faculty of Medicine, Chiang Mai University, Chiang Mai 50200, Thailand

[11]Mae Sai District Hospital, Mae Sai, Chiang Rai 57130, Thailand

Supplementary Online Content

**Table S1**	Univariable Predictors of Treatment Failure in PD-Related Peritonitis	S3
**Table S2**	Multivariable Predictors and Goodness-of-Fit for Sequential Models of Treatment Failure among PD Patients with Peritonitis	S6
**Table S3**	Performances of a Risk Score Predicting Treatment Failure among PD-Related Peritonitis	S7
**Table S4**	Sensitivity Analysis of Predicted Risk Score	S8
**Figure S1**	Study Flow on the Selection of Eligible Patients for Clinical Prediction Rule	S9
**Figure S2**	Score Predicted and Observed Risk of Treatment Failure in PD-Related Peritonitis by Each Total Score	S10
**Figure S3**	The AuROC Curve of the Prediction Score of Treatment Failure in PD-Related Peritonitis according to the Internal Validation Cohort	S11

Table S1. Univariable Predictors of Treatment Failure in PD-Related Peritonitis (n=855 episodes)

Predictors	β-Coefficients (SE)	Crude OR (95% CI)	*P* Value	AuROC (95% CI)
Gender				
Male		1.00 (Reference)		
Female	0.10 (0.16)	1.10 (0.80 – 1.51)	0.549	0.51 (0.47 – 0.55)
Age, years				
<65		1.00 (Reference)		
≥65	-0.04 (0.17)	0.96 (0.69 – 1.33)	0.811	0.50 (0.46 – 0.53)
Primary cause of ESKD				
Hypertensive nephrosclerosis		1.00 (Reference)		
Diabetic nephropathy	-0.14 (0.19)	0.86 (0.59 – 1.26)	0.455	0.47 (0.43 – 0.52)
Glomerulonephritis	-0.01 (0.35)	0.99 (0.50 – 1.97)	0.977	
Others/unknown	-0.28 (0.22)	0.76 (0.50 – 1.16)	0.206	
Hypertension				
No		1.00 (Reference)		
Yes	0.46 (0.28)	1.59 (0.93 – 2.73)	0.092	0.52 (0.50 – 0.55)
Diabetes mellitus				
No		1.00 (Reference)		
Yes	0.32 (0.17)	1.38 (1.00 – 1.92)	0.052	0.54 (0.50 – 0.58)
CAD				
No		1.00 (Reference)		
Yes	-0.23 (0.34)	0.79 (0.41 – 1.55)	0.498	0.49 (0.47 – 0.51)
CHF				
No		1.00 (Reference)		
Yes	-0.13 (0.32)	0.88 (0.47 – 1.65)	0.687	0.50 (0.48 – 0.52)
CVD				
No		1.00 (Reference)		
Yes	0.56 (0.33)	1.74 (0.91 – 3.33)	0.092	0.51 (0.49 – 0.53)
Malignancy				
No		1.00 (Reference)		
Yes	-0.49 (0.71)	0.61 (0.15 – 2.44)	0.486	0.49 (0.48 – 0.50)
Chronic hepatitis B				
No		1.00 (Reference)		
Yes	0.18 (1.01)	1.19 (0.16 – 8.71)	0.862	0.50 (0.49 – 0.51)
Chronic hepatitis C				
No		1.00 (Reference)		
Yes	-1.64 (1.10)	0.19 (0.02 – 1.68)	0.137	0.49 (0.48 – 0.50)
Mobility				
Independent walker		1.00 (Reference)		
Assisted walker	0.65 (0.45)	1.92 (0.80 – 4.59)	0.145	0.53 (0.50 – 0.56)
Chair-bound/ bedridden	0.32 (0.22)	1.37 (0.89 – 2.11)	0.162	

Abbreviations: AuROC, area under the receiver operating characteristic; CAD, coronary artery disease; CHF, chronic heart failure; CI, confidence interval; CVD, cerebrovascular disease; ESKD, end-stage kidney disease; OR, odds ratio; PD, peritoneal dialysis; SE, standard error.

Table S1. Univariable Predictors of Treatment Failure in PD-Related Peritonitis (n=902 episodes) (Continued)

**Predictors**	**β-Coefficients (SE)**	**Crude OR (95% CI)**	***P* Value**	**AuROC (95% CI)**
Reimbursement scheme				
UCS by NHSO		1.00 (Reference)		
CSMBS	-0.26 (0.25)	0.77 (0.47 – 1.27)	0.310	0.48 (0.46 – 0.51)
SSS/others	-0.05 (0.46)	0.95 (0.38 – 2.34)	0.911	
PD modality				
CAPD		1.00 (Reference)		
APD	-0.72 (0.51)	0.48 (0.18 – 1.32)	0.157	0.49 (0.48 – 0.50)
PD with assistance				
Alone/with family		1.00 (Reference)		
With others	1.50 (0.71)	4.48 (1.11 – 18.14)	0.035	0.50 (0.50 – 0.51)
Dialysis duration, years				
<1		1.00 (Reference)		
1 – 2	-0.04 (0.21)	0.96 (0.63 – 1.46)	0.849	0.50 (0.46 – 0.54)
>2	-0.00 (0.18)	1.00 (0.70 – 1.42)	0.996	
Systolic BP, mmHg				
<90	1.45 (0.27)	4.28 (2.50 – 7.33)	<0.001	0.56 (0.53 – 0.58)
≥90		1.00 (Reference)		
Diastolic BP, mmHg				
<60	0.24 (0.26)	1.27 (0.76 – 2.11)	0.365	0.51 (0.49 – 0.53)
≥60		1.00 (Reference)		
Fever (°C)				
≤38.9		1.00 (Reference)		
>38.9	0.68 (0.31)	1.98 (1.08 – 3.64)	0.028	0.52 (0.50 – 0.54)
Abdominal pain				
No		1.00 (Reference)		
Yes	-0.18 (0.19)	0.83 (0.57 – 1.21)	0.337	0.48 (0.45 – 0.52)
Diarrhoea				
No		1.00 (Reference)		
Yes	0.07 (0.18)	1.07 (0.76 – 1.52)	0.696	0.51 (0.47 – 0.54)
Constipation				
No		1.00 (Reference)		
Yes	-0.21 (0.33)	0.81 (0.42 – 1.54)	0.515	0.49 (0.48 – 0.51)
Catheter leak				
No		1.00 (Reference)		
Yes	0.73 (0.44)	2.07 (0.87 – 4.95)	0.100	0.51 (0.50 – 0.53)
Cloudy dialysate fluid				
No		1.00 (Reference)		
Yes	-0.52 (0.29)	0.60 (0.34 – 1.06)	0.076	0.48 (0.46 – 0.50)
Disease severity score				
<3		1.00 (Reference)		
≥3	0.67 (0.28)	1.95 (1.12 – 3.39)	0.019	0.53 (0.50 – 0.55)

Abbreviations: APD, automated peritoneal dialysis; AuROC, area under the receiver operating characteristic; BP, blood pressure; CAPD, continuous ambulatory peritoneal dialysis; CI, confidence interval; CSMBS, the Civil Servant Medical Benefit Scheme; NHSO, the National Health Security Office; OR, odds ratio; PD, peritoneal dialysis; SE, standard error; SSS, the Social Security Scheme; UCS, the Universal Coverage Scheme.

Table S1. Univariable Predictors of Treatment Failure in PD-Related Peritonitis (n=902 episodes) (Continued)

**Predictors**	**β-Coefficients (SE)**	**Crude OR (95% CI)**	***P* Value**	**AuROC (95% CI)**
Serum albumin				
<3	0.07 (0.22)	1.10 (0.86 – 1.71)	0.822	0.54 (0.51-0.60)
≥3		1.00 (Reference)		
Dialysate leucocyte count on day 1 >1,350/mm^3				
No		1.00 (Reference)		
Yes	0.61 (0.17)	1.85 (1.32 – 2.59)	<0.001	0.58 (0.54 – 0.62)
Dialysate leucocyte count on day 3 – 4 >1,000/mm^3				
No		1.00 (Reference)		
Yes	2.18 (0.19)	8.84 (6.07 – 12.87)	<0.001	0.72 (0.68 – 0.75)
Dialysate leucocyte count on day 5 >100/mm^3				
No		1.00 (Reference)		
Yes	4.04 (0.26)	57.10 (34.52 – 94.47)	<0.001	0.88 (0.86 – 0.91)
Causative organism				
Gram-positive only[a]		1.00 (Reference)		
MRSA	2.95 (0.64)	19.13 (5.43 – 67.38)	<0.001	0.58 (0.54 – 0.62)
Gram-negative only[b]	1.12 (0.32)	3.05 (1.62 – 5.74)	0.001	
Acinetobacter spp.	1.92 (0.37)	6.83 (3.33 – 14.03)	<0.001	
Pseudomonas spp.	2.30 (0.47)	10.02 (3.96 – 25.37)	<0.001	
Fungi	4.47 (0.62)	87.47 (26.10 – 293.10)	<0.001	
Mycobacterial	3.20 (0.84)	24.60 (4.77 – 126.91)	<0.001	
Polymicrobial	2.15 (0.30)	8.56 (4.74 – 15.43)	<0.001	
Culture negative	0.80 (0.22)	2.22 (1.43 – 3.43)	<0.001	
Antimicrobial for empirical therapy				
First generation cephalosporin-based regimen		1.00 (Reference)		
Glycopeptide-based regimen	0.48 (0.19)	1.61 (1.10 – 2.34)	0.013	0.57 (0.53 – 0.61)
Others regimen	0.81 (0.26)	2.24 (1.36 – 3.72)	0.002	

[a]Excluding MRSA; [b]Excluding *Acinetobacter spp.* and *Pseudomonas spp.*

Abbreviations: AuROC, area under the receiver operating characteristic; CI, confidence interval; MRSA, Methicillin-resistant *Staphylococcus aureus*; OR, odds ratio; PD, peritoneal dialysis; SE, standard error.

Table S2. Multivariable Predictors and Goodness of Fit for Sequential Models of Treatment Failure among PD Patients with Peritonitis

**Variable**	**Adjusted OR (95% CI)**				
	**Model 1**	**Model 2**	**Model 3**	**Model 4**	**Model 5**
Age ≥65 years	1.08 (0.62 – 1.88)[a]				
Female sex	0.88 (0.53 – 1.46)[a]				
Diabetes	1.82 (1.07 – 3.09)	1.80 (1.06 – 3.06)	1.76 (1.04 – 2.98)	1.81 (1.09 – 3.01)	1.52 (1.04 – 2.22)
Systolic BP <90 mmHg	3.79 (1.34 – 10.73)	3.72 (1.31 – 10.56)	3.77 (1.29 – 11.04)	4.36 (1.72 – 11.09)	3.64 (1.93 – 6.84)
Dialysate leucocyte count on day 1 >1,350/mm^3	0.68 (0.39 – 1.20)[a]	0.68 (0.38 – 1.19)[a]			
Dialysate leucocyte count on day 3–4 >1,000/mm^3	2.25 (1.23 – 4.12)	2.24 (1.22 – 4.10)	2.33 (1.31 – 4.12)	2.52 (1.50 – 4.23)	8.94 (6.05 – 13.20)
Dialysate leucocyte count on day 5 >100/mm^3	51.78 (27.73 – 96.70)	51.93 (27.86 – 96.79)	45.26 (24.46 – 83.74)	43.64 (25.69 – 74.16)	
Causative organism[b]					
MRSA	2.15 (0.30 – 15.40)[a]	2.34 (0.33 – 16.78)[a]	2.52 (0.36 – 17.73)[a]		
Gram-negative only[c]	1.78 (0.61 – 5.19)[a]	1.81 (0.63 – 5.20)[a]	1.74 (0.61 – 4.92)[a]		
Acinetobacter spp.	4.91 (0.81 – 29.82)[a]	4.80 (0.79 – 29.06)[a]	6.35 (1.13 – 35.60)		
Pseudomonas spp.	3.32 (0.97 – 11.29)[a]	3.21 (0.95 – 10.90)[a]	3.14 (1.00 – 9.84)		
Fungi	65.48 (8.05 – 532.57)	65.14 (7.94 – 534.30)	73.18 (8.59 – 623.10)		
Mycobacterial	4.54 (0.50 – 40.85)[a]	4.28 (0.50 – 36.36)[a]	4.64 (0.58 – 37.07)[a]		
Polymicrobial	4.99 (2.13 – 11.70)	4.96 (2.14 – 11.48)	4.85 (2.10 – 11.23)		
Culture negative	1.35 (0.77 – 2.38)[a]	1.36 (0.77 – 2.39)[a]	1.36 (0.77 – 2.40)[a]		
C statistic[d]	0.94 (0.92 – 0.96)	0.94 (0.92 – 0.96)	0.94 (0.92 – 0.96)	0.92 (0.89 – 0.94)	0.76 (0.72 – 0.80)
Akaike Information Criterion[d]	437.47	433.80	444.05	482.03	760.11
P Value[e]	0.512	0.160		<0.001	<0.001

Abbreviations: BP, blood pressure; CI, confidence interval; MRSA, Methicillin-resistant *Staphylococcus aureus*; OR, odds ratio; PD, peritoneal dialysis.

[a]Odds ratios with *P* value > 0.05; all other odds ratios are significant (*P* value < 0.05).

[b]Compared to gram-positive only (excluding MRSA).

[c]Excluding *Acinetobacter spp.* and *Pseudomonas spp.*

[d]Null values for C statistic is 0.50. Higher values for C statistic and lower values Akaike Information for Criterion indicate better models.

[e]*P* values are for comparison of C statistics between model 3 and other models.

Table S3. Performances of a Risk Score Predicting Treatment Failure among PD-Related Peritonitis

Risk Score Points	Observed Incidence of Treatment Failure (in %)	Sensitivity, % (95% CI)	Specificity, % (95% CI)	LHR+ (95% CI)	LHR- (95% CI)	PPV, % (95% CI)	NPV, % (95% CI)
≥1	39.5	96.6 (93.0 – 98.6)	50.1 (46.0 – 54.2)	1.93 (1.78 – 2.10)	0.07 (0.03 -0.14)	39.5 (35.2 – 44.0)	97.7 (95.4 – 99.1)
≥1.5	56.4	93.1 (88.7 – 96.2)	75.7 (72.1 – 79.1)	3.83 (3.31 – 4.44)	0.09 (0.05 – 0.15)	56.4 (50.9 -61.8)	97.0 (95.0 -98.4)
≥2.5	60.8	92.6 (88.1 – 95.8)	79.9 (76.4 – 83.0)	4.60 (3.90 – 5.42)	0.09 (0.06 – 0.15)	60.8 (55.2 – 66.3)	97.0 (95.1 – 98.3)
≥3.5	66.3	91.1 (86.3 – 94.7)	84.4 (81.2 – 87.2)	5.83 (4.82 – 7.05)	0.10 (0.07 – 0.16)	66.3 (60.4 – 71.8)	96.6 (94.6 – 98.0)
≥4	68.0	90.1 (85.2 – 93.9)	85.7 (82.6 – 88.4)	6.30 (5.15 – 7.70)	0.12 (0.08 – 0.18)	68.0 (62.1 – 73.6)	96.3 (94.3 – 97.7)
≥6.5	68.8	90.1 (85.2 – 93.9)	86.2 (83.2 – 88.8)	6.53 (5.32 – 8.01)	0.11 (0.08 – 0.17)	68.8 (62.9 – 74.3)	96.3 (94.3 – 97.7)
≥7.5	74.6	73.9 (67.3 – 79.8)	91.5 (89.0 – 93.6)	8.71 (6.61 – 11.50)	0.28 (0.23 – 0.36)	74.6 (68.0 – 80.5)	91.2 (88.7 – 93.3)
≥8	79.3	58.6 (51.5 – 65.5)	94.8 (92.8 – 96.5)	11.4 (7.91 – 16.30)	0.44 (0.37 – 0.51)	79.3 (72.0 – 85.5)	87.2 (84.3 – 89.6)
≥9	87.3	30.5 (24.3 – 37.4)	98.5 (97.2 – 99.3)	20.4 (10.3 – 40.3)	0.70 (0.64 – 0.77)	87.3 (77.3 – 94.0)	80.8 (77.7 – 83.6)
≥10	89.5	8.4 (5.0 – 13.1)	99.7 (98.8 – 100.0)	25.20 (5.86 – 108.00)	0.92 (0.88 – 0.96)	89.5 (66.9 – 98.7)	76.3 (73.2 – 79.2)
≥10.5	82.2	7.4 (4.2 – 11.9)	99.7 (98.9 – 100.0)	22.20 (5.12 – 96.30)	0.93 (0.89 – 0.97)	88.2 (63.6 – 98.5)	76.1 (73.0 – 79.1)
≥11.5	80.0	2.0 (0.5 – 5.0)	99.8 (99.1 – 100.0)	11.80 (1.33 – 105.00)	0.98 (0.96 – 1.00)	80.0 (58.4 – 99.5)	75.1 (71.9 – 78.1)

Abbreviations: CI, confidence interval; LHR+, likelihood ratio of positive; LHR-, likelihood ratio of negative; NPV, negative predictive value; PPV, positive predictive value.

Table S4. Sensitivity Analysis of Predicted Risk Score

**Type of Sensitivity Analysis**	**N, Episodes**	**C statistic (95% CI)**	***P* for Hosmer-Lemshow statistic**
The multiple imputation analysis of the predicted risk score	855	0.94 (0.92 – 0.95)	1.000
Restricting analysis to outcome which defined as only catheter removal	762	0.92 (0.90 – 0.94)	0.864
Excluding MRSA and *Pseudomonas spp.* peritonitis	779	0.91 (0.89 – 0.94)	0.949
Excluding *Mycobacterium* and fungal peritonitis	762	0.93 (0.90 – 0.95)	0.672
Excluding episodes with culture negative	520	0.90 (0.87 – 0.93)	0.827

Abbreviations: CI, confidence interval; MRSA, Methicillin-resistant *Staphylococcus aureus*.

Figure S1. Study Flow on the Selection of Eligible Patients for Clinical Prediction Rule.

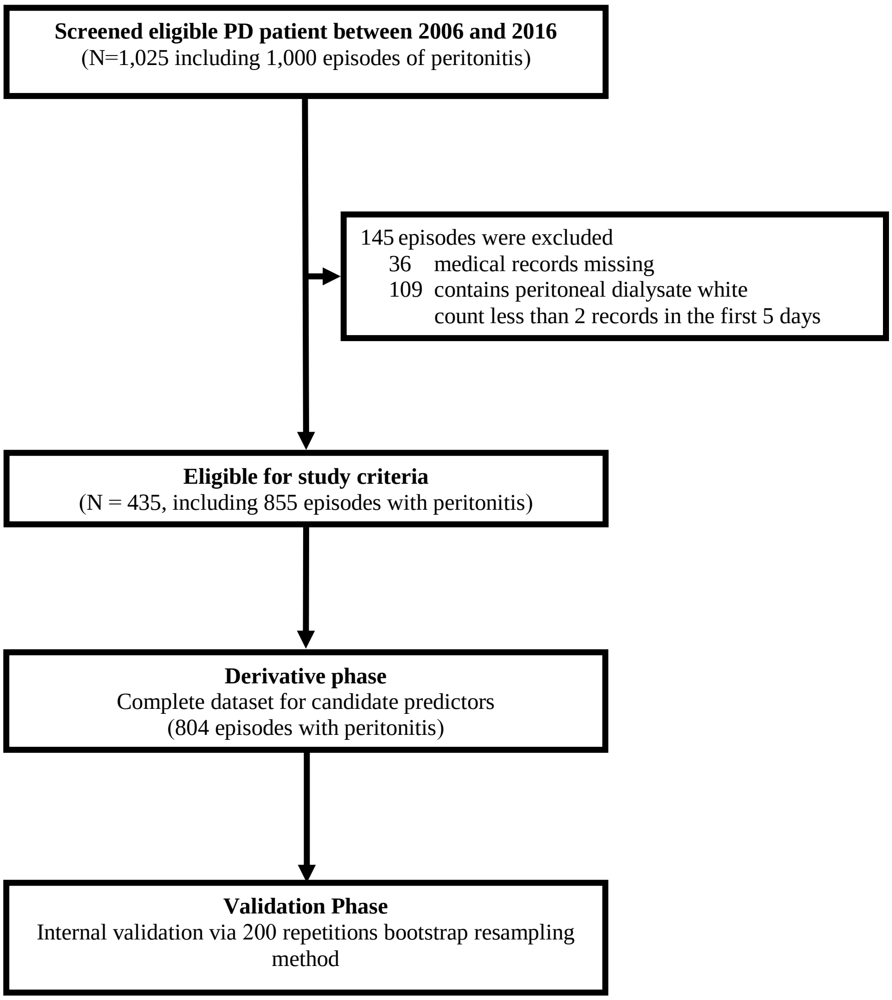

Abbreviations: PD, peritoneal dialysis.

Figure S2. Score Predicted and Observed Risk of Treatment Failure in PD-related Peritonitis by Each Total Score.

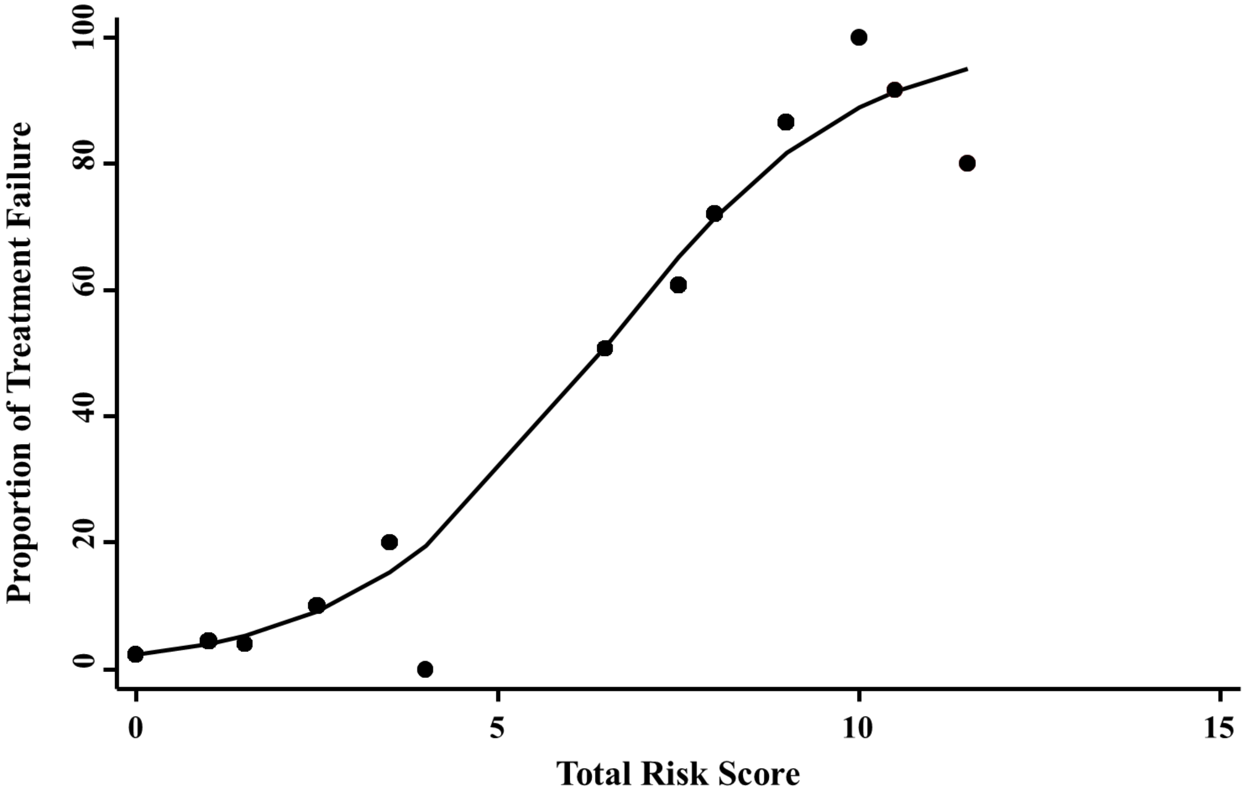

Note: Logistic estimated risk (solid line) against actual risk (dots) for treatment failure in PD-related peritonitis by each total score.
Abbreviations: PD, peritoneal dialysis.

Figure S3. The AuROC Curve of the Prediction Score of Treatment Failure in PD-Related Peritonitis according to the Internal Validation Cohort.

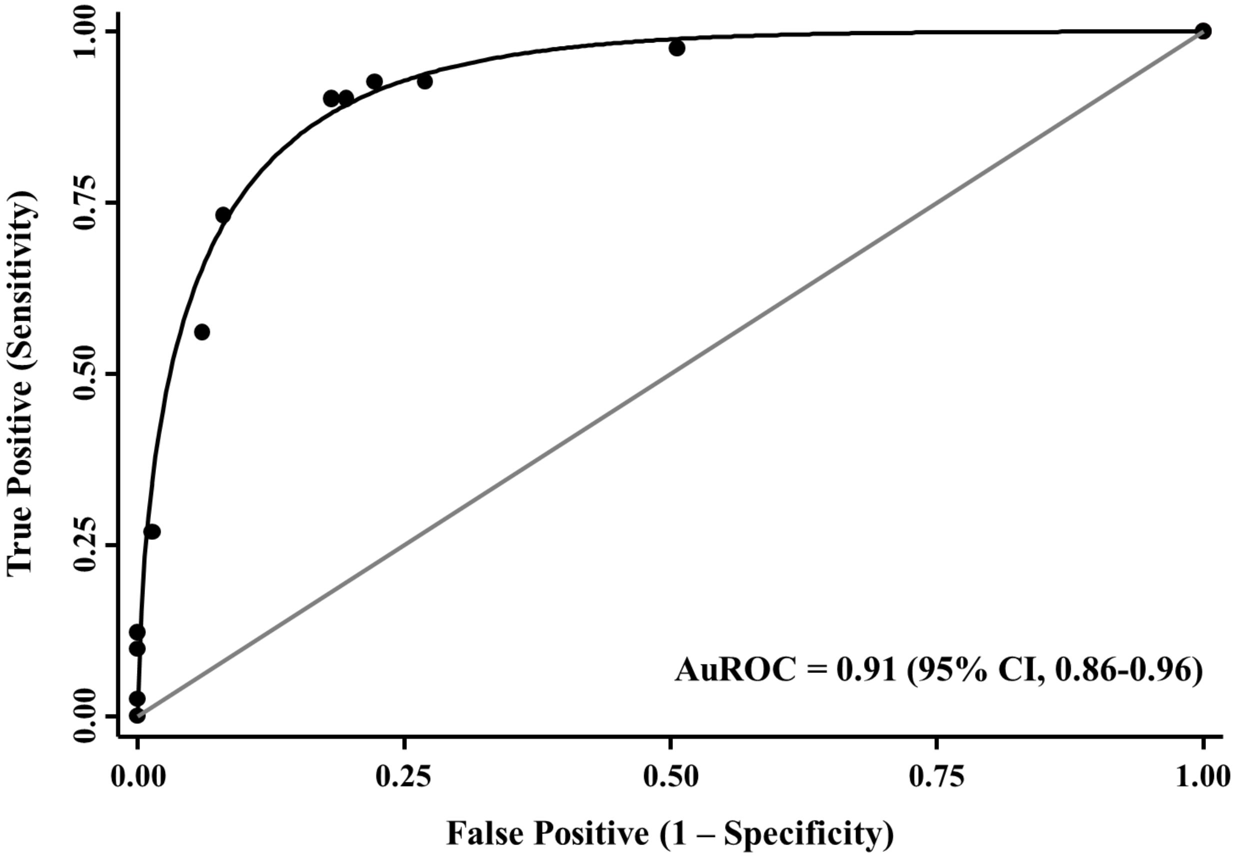

Abbreviations: AuROC, area under the receiver operating characteristic; CI, confidence interval, PD, peritoneal dialysis.
